# Fetal Bovine Serum Modulates Primary Human Cell Phenotypes, Endothelial Barrier Function, Vasculogenesis, and Angiogenesis in a Sex-Specific Manner

**DOI:** 10.1007/s12195-025-00860-3

**Published:** 2025-09-05

**Authors:** Ashley Martier, G. Wills Kpeli, Keefer Boone, Isabella R. Posey, Mark J. Mondrinos

**Affiliations:** 1 Department of Biomedical Engineering, Tulane University School of Science & Engineering, 6823 St. Charles Avenue, New Orleans, LA 70118 USA; 2 Tulane Center of Excellence in Sex-based Precision Medicine, New Orleans, LA USA; 3 Department of Physiology, Tulane University School of Medicine, New Orleans, LA USA; 4 Tulane Cancer Center, Louisiana Cancer Research Center, New Orleans, LA USA

**Keywords:** Tissue engineering, Microphysiological systems, Endothelial cells, Fetal bovine serum, Sex differences, Sex hormones

## Abstract

**Purpose:**

Sex differences in cellular biology significantly influence cell responses in culture. Yet, the sex-specific effects of culture reagents such as fetal bovine serum (FBS) remain understudied. Increased adoption of cell-based models such as microphysiological systems (MPS) as replacements for animal models demands a greater understanding sex-specific responses to common media formulations. This study examined the effects of FBS and hormone-free charcoal-stripped serum (CSS) on male (XY) and female (XX) cells in 2D and 3D MPS culture models to demonstrate profound sex-specificity in bioassays and inform the development of future sex-specific cell culture protocols and methods.

**Methods:**

Primary human endothelial cells and fibroblasts from multiple organ sources were cultured in 2D and in 3D MPS models. Cells were cultured with either FBS or CSS. Endothelial specific gene expression, cytoskeletal spreading, and cell cycle status were analyzed in 2D culture. Vascular network formation, macromolecular leakage, and directional angiogenic sprouting were assessed in 3D MPS models.

**Results:**

FBS promoted significant upregulation of genes associated with endothelial function in XX endothelial cells, but the same gene clusters were downregulated in XY cells. FBS increased cytoskeletal spreading and cell cycle participation of XX endothelial cells and fibroblasts relative to culture with CSS. Conversely, culture with CSS increased these 2D metrics in XY cells. Measurement of 40 kDa FITC-dextran leakage in a single vessel MPS model revealed that culture with FBS significantly decreased XX endothelial barrier permeability relative to culture with CSS. In line with 2D assays, CSS conversely enhanced XY endothelial barrier permeability relative to culture with FBS. Culture with FBS increased metrics of vasculogenesis in XX tissues relative to CSS cultures, whereas prolonged cultured in CSS supported vasculogenesis in XY models. MPS angiogenesis assays revealed increased sprouting in XX tissues cultured with FBS, while only minimal sprouting was observed in all other conditions.

**Conclusions:**

FBS imparted significant sex-specific effects on the gene expression patterns, morphology, and cell cycle status of human endothelial cells and fibroblasts in 2D culture. Sex-specific effects measured in 2D culture assays carried over to 3D MPS assays of endothelial barrier function, vasculogenesis, and angiogenesis. Notably, FBS significantly enhanced XX cell functions relative to XY cells in all 2D and 3D MPS assays. Thus, accounting for the sex-specific effects of culture media components will be imperative to improve reproducibility and translational relevance of MPS in preclinical research.

**Supplementary Information:**

The online version contains supplementary material available at 10.1007/s12195-025-00860-3.

## Introduction

The biological sex of cultured cells is increasingly recognized as a critical factor that modulates phenotypes and therefore functional readouts in preclinical research. The National Institutes of Health (NIH) have issued guidance that requires sex be considered in all preclinical research, including in cell culture applications [1, 2]. This mandate has increased awareness that cell sex significantly impacts behavior in culture and reporting cell sex in publications has increased accordingly [2–9]. Sex herein refers to the biological phenotypes imparted by sex chromosome composition (i.e. female cells contain XX chromosomes and male cells contain XY chromosomes), gametes, and sex hormones [10]. Beyond the historical emphasis on sex chromosomes and hormones in endocrinology, emerging research highlights the crucial roles of sex-specific patterns of paracrine factors and mechanical properties of the cellular environment in modulating sex-specific phenotypes [11–16]. Gender is related to the impacts of societal constructs and human self-perception that interplay with sex to influence physiology, disease, and clinical outcomes [10]. While recognizing the interdependence of sex and gender in the context of medicine, our studies and discussion thereof focus on accounting for the role of cell sex in areas of biotechnology including cell culture, tissue engineering, and microphysiological systems [4, 10, 17].

Although cell sex is increasing reported in preclinical research, many studies still fail to consider the critical interplay of sex hormones and sex chromosomes in the manifestation of sex-specific phenotypes. Fetal bovine serum (FBS), a central component of many culture media formulations, has been shown to display estrogenic effects that can significantly modulate the growth and proliferation of cells [18–20]. Anticipating these effects is complicated by natural variation between sources and individual lots of FBS [21–23]. FBS may be charcoal stripped to remove hormones. Charcoal-stripped serum (CSS) has traditionally been used to eliminate hormone effects or as a base for creating culture medium with defined sex hormone concentrations [19, 24].

While preclinical science has relied largely on the use of animal models, a shift to cell culture-based models is occurring with the growth of fields such as microphysiological systems (MPS) [25–27]. The FDA Modernization Act 2.0 has allowed for the use of MPS and other tissue engineered models in preclinical testing as ‘new approach methodologies’ (NAM) that can serve as alternatives to animal models [28]. However, continued research and development is needed to maximize the physiological relevance and translational value of these models. Factors such as cell sex and sex-specific effects of culture medium must be considered at all stages from system development to study design. Previous work has shown that cells used in MPS models of the vasculature respond to sex hormones in a sex-specific manner [29, 30]. Further evaluation of sex-specific responses to common culture conditions, for example the presence of FBS, must be explored in greater depth to inform interpretation of donor variability in cell culture assays and MPS and begin establishing a framework of sex-based culture protocols [31].

Herein, we report the sex-specific effects of FBS and CSS in a range of 2D culture and 3D MPS assays. Compared to CSS, FBS increased proliferation and cytoskeletal spreading in XX cultures of primary endothelial cells and fibroblasts and decreased the same metrics in XY cultures. An endothelial barrier MPS showed that FBS enhanced endothelial barrier function in XX devices compared to XX devices cultured in CSS, while XY devices exhibited improved barrier function when cultured with CSS. An MPS bulk vasculogenesis assay revealed that FBS enhances XX vasculogenesis compared to culture with CSS, while switching to CSS enhanced XY vasculogenesis. MPS angiogenesis assays showed that FBS supported VEGF gradient-induced sprouting in XX tissues with only minimal sprouting in all other conditions tested. Collectively, this work aims to elucidate the effects of FBS on male and female cells in a range of 2D and 3D MPS bioassays and motivate the development of sex-specific culture protocols that can improve physiological relevance.

## Materials and Methods

### Experimental Design

**Fig. 1:**
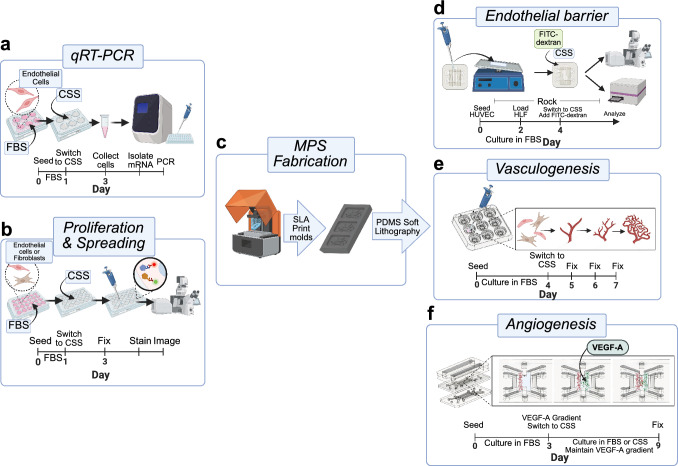
Overall study design. **a** Cells were grown in 6-well plates in experimental conditions for 48 h before collection, mRNA isolation, and PCR. **b** For immunofluorescence assays of spreading and proliferation, cells were grown in experimental conditions for 48 h, fixed, stained and imaged. **c** For MPS studies, molds were manufactured using a SLA printer and MPS devces were then fabricated via PDMS soft lithography. **d** HUVEC and HLF were loaded into 4-layer PDMS devices for endothelial barrier assays. Half remained in FBS, and half were transferred to CSS. Endothelial patency and permeability were assessed by measuring FITC-dextran transport across the endothelial barrier. **e** HUVEC and HLF embedded in collagen I and fibrin co-gels were loaded into PDMS arrays for vasculogenesis assays and networks formed for 4 days in FBS. At day 4, half of devices were transferred to CSS (experimental) and half remained in FBS (control). Cohorts of devices were fixed on day 4, day 5, day 6, and day 7. **f** The same hydrogel-embedded mixture of HUVEC and HLF were loaded into the left channel of angiogenesis MPS devices on day 0 and allowed to form networks in FBS for 3 days. At day 3, half of the devices were transferred to CSS, and a VEGF-A gradient established across all devices to induce directional angiogenic sprouting. Devices were then cultured in either CSS or FBS for 6 days. VEGF-A gradients were replenished by reloading the media channels every 24 h until fixation on day 9. Created with Biorender.com.

**Fig. 2:**
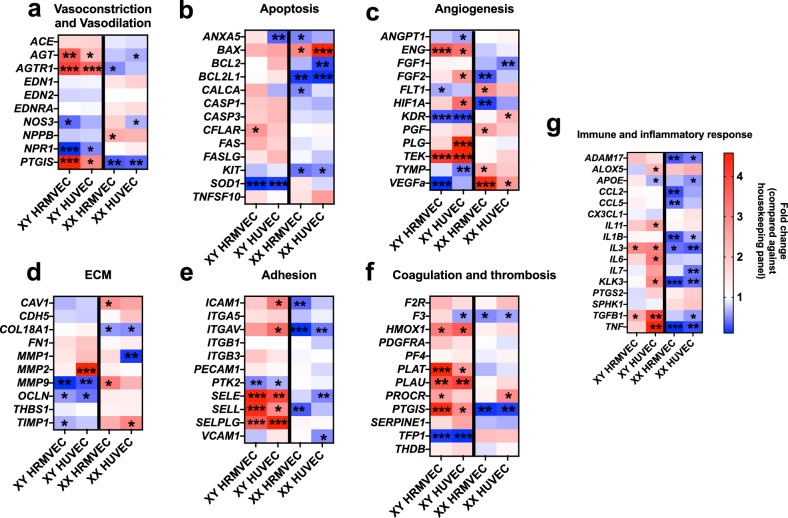
FBS induces sex-specific endothelial gene expression responses in 2D culture. RT-qPCR results from 96 gene Qiagen Endothelial Gene arrays following culture of HUVEC and HRMVEC in CSS- or FBS-containing media. Cells were exposed to specified conditions for 48 h before collection. Thick black vertical lines separate XY (left) and XX (right) datasets. Data are displayed as expression in FBS compared to the same cells grown in CSS. Genes were clustered in groups associated with vasoconstriction and vasodilation (**a**), apoptosis (**b**), angiogenesis (**c**), ECM production and remodeling (**d**), adhesion (**e**), coagulation (**f**), and inflammatory responses (**g**). White cells represent a fold change of 1 (i.e. no change between FBS and CSS) with blue showing downregulation and red showing upregulation on the indicated scale. Compared to housekeeping panel (standardized to fold change = 1) in same cells grown in CSS via two-way ANOVA with Bonferroni correction. * = p < 0.05, ** = p < 0.01, *** = p < 0.001. Sex main effect: p < 0.001, Serum main effect: p < 0.001. n = 3 donors per sex for HRMVEC, 4 donors per sex for HUVEC; 3 technical replicates per donor.

**Fig. 3:**
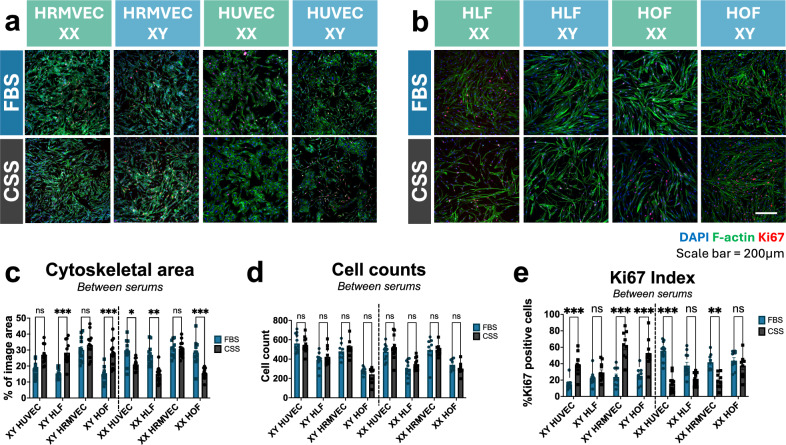
Sex-specific effects of FBS and CSS on cytoskeletal spreading and Ki67 indexes. **a:** Representative micrographs of XX and XY HUVEC and HRMVEC grown in either FBS or CSS for 48 h stained for DAPI (blue), F-actin (green), and Ki67 (red). Scale bar = 200 μm. **b** Representative micrographs of XX and XY HLF and HOF grown in either FBS or CSS for 48 h stained for DAPI (blue), F-actin (green), and Ki67 (red). Scale bar = 200 μm. **c** Cytoskeletal area coverage determined by analysis of F-actin staining. **d:** Cell counts determined by analysis of DAPI staining of cell nuclei. **e:** Ki67 indexes calculated as the percentage of Ki67-positive cells. * = p < 0.05, ** = p < 0.01, *** = p < 0.001. All statistics were analyzed via two-way ANOVA in GraphPad Prism. Cytoskeletal area: sex main effect = p < 0.001, serum main effect p = 0.55, interaction effect = p < 0.001. Cell count: sex main effect = p < 0.001, serum main effect = p = 0.31, interaction effect = p = 0.41. Ki67 index: sex main effect = p < 0.001, serum main effect = p = 0.51, interaction effect = p < 0.001. Error bars represent SEM. n = 3 donors per sex for HOF and HRMVEC; 4 donors per sex for HLF and HUVEC. 3 wells per donor, 3 images per well. Dots represent per well average.

**Fig. 4:**
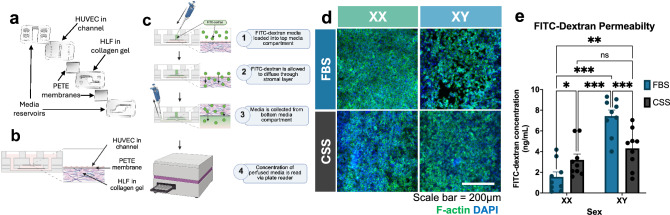
Sex-specific effects of FBS and CSS on endothelial barrier formation and permeability. **a** Exploded view CAD render of four-layer PDMS device composed of endothelial barrier layer and underlying stromal compartment separated by polyester (PETE) membranes from top and bottom media reservoirs. **b** Side view of CAD device with demonstrated loading of HUVEC and HLF. **c** Schematic detailing steps of FITC-dextran permeability assay. Schematics created using Biorender.com. **d** HUVEC monolayers grown in devices were cultured with CSS or FBS for 48 h prior to fixation and stained for DAPI (blue) and F-actin (green). Scale bar = 200 μm. **e** FITC-dextran permeability assay. Data represent the concentration (ng/ml) of 40 kDa FITC-dextran transferred to the collection channel medium. * = p < 0.05, ** = p < 0.01, *** = p < 0.001. Analyzed via two-way ANOVA in GraphPad Prism. Permeability sex main effect = p = 0.002, permeability serum main effect = p = 0.37, permeability interaction effect = p = 0.02. Error bars represent SEM. n = 6 devices per condition; 3 HUVEC donors per sex, 2 devices per donor. Dots represent one device.

**Fig. 5:**
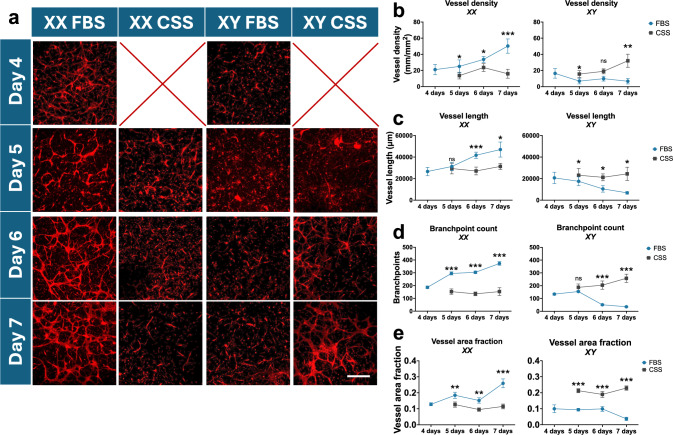
Sex-specific effects of FBS and CSS on bulk tissue vasculogenesis. **a** Representative micrographs of XX and XY vascular networks cultured in hormone-rich (FBS) and hormone-depleted (CSS) media and fixed at days 4, 5, 6, and 7 in culture. Endothelial cells were labeled by staining with UEA-1 (red). All devices were kept in FBS through day 4. Scale bar = 200 μm. **b–e** Morphometric analysis of vascular network formation; vessel density (**b**), vessel length (**c**), branchpoint count (**d**), and vessel area fraction (**e**) at days 4–7 in FBS and days 5-7 in CSS. * = p < 0.05, ** = p < 0.01, *** = p < 0.001. All statistics were analyzed via two-way ANOVA in GraphPad Prism. Vessel density: sex main effect = p < 0.001, serum main effect = p < 0.001, interaction effect = p < 0.001. Vessel length: sex main effect = p = 0.37, serum main effect = p < 0.001, interaction effect = p < 0.001. Branchpoint count: sex main effect = p < 0.001, serum main effect = p < 0.001, interaction effect = p < 0.001. Vessel area fraction: sex main effect = p = 0.04, serum main effect = p = 0.09, interaction effect = p < 0.001. Error bars represent SEM. n = 6 devices per condition per sex; 3 randomized pairs of HUVEC and HLF were used per sex, 2 devices per pairing.

**Fig. 6:**
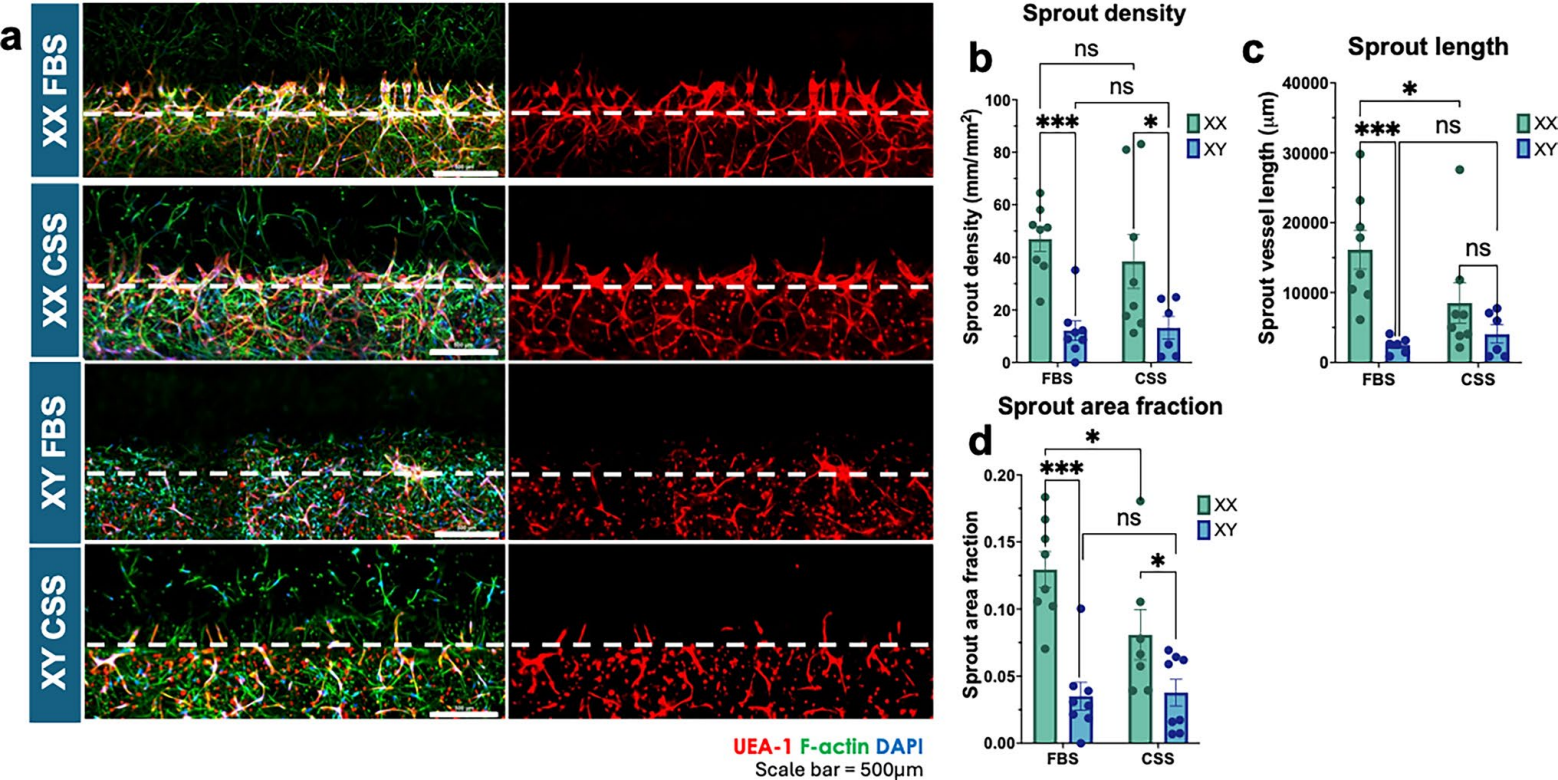
Sex-specific effects of FBS and CSS on VEGF-gradient induced sprouting angiogenesis. **a** Representative 3D stitched LSCM of Male (XY) and Female (XX) sex-matched vascular network and sprouting after 10 days in hormone-rich (FBS) and hormone-depleted (CSS) culture. Endothelial cells are labelled with UEA-1 lectin (red). F-Actin in all cells is labelled with phalloidins (green), fibroblasts are green only. Nuclei of all cells are labelled with DAPI (blue). Scale bar = 500um. White dashed lines represent boundary between vascular margin and sprout angiogenesis regions. **b**–**d** Morphometric analysis of sprout vessel characteristics. * = p < 0.05, ** = p < 0.01, *** = p < 0.001. All statistics were analyzed via two-way ANOVA in GraphPad Prism. Sprout density: sex main effect = p = 0.58, serum main effect = p < 0.001, interaction effect = p = 0.48. Sprout length: sex main effect = p = 0.23, serum main effect = p = 0.001, interaction effect = p = 0.07. Sprout area fraction: sex main effect = p = 0.09, serum main effect = p < 0.001, interaction effect = p = 0.06. Error bars represent SEM. n = 5 devices per condition per sex; 2 randomized pairs of HUVEC and HLF were used per sex, 2-3 technical replicates per pairing. Dots represent individual devices.

For 2D immunofluorescence assays and RT-qPCR studies (Figures 1, 2, 3), all donors (see Figure S1) were utilized to maximize consistency of interpreting results across assays. For the endothelial barrier permeability (Figure 4) and vasculogenesis MPS models (Figure 5), HLF and HUVEC were randomly assigned into sex-matched pairs, and 3 pairs were used for each experiment. For the angiogenesis MPS model (Figure 6), 2 randomized donor pairs per sex were used for each experiment. FBS and CSS lots used throughout experimentation were randomized from the lots characterized by estradiol ELISA analysis (Figure S2).

### Cell Culture

XX and XY primary human lung fibroblasts (HLF, ATCC), human umbilical vein endothelial cells (HUVEC), human ocular fibroblasts (HOF), and human retinal microvascular endothelial cells (HRMVEC) were used in these studies. 4 donors per sex were used for HUVEC and HLF, and 3 donors per sex were used for HOF and HRMVEC. Cell suppliers, catalog numbers, and available donor information for each lot are included in Supplemental Figure 1. All cells other than embryonic HUVEC were sourced from donors aged 14–45 years. HUVEC and HRMVEC were expanded in VascuLife VEGF Endothelial Complete Medium (Lifeline Cell Technology, LL-0003). HLF and HOF were expanded in Fibroblast S2 Fibroblast Complete Medium (Lifeline Cell Technology, LL-0011). For culture in FBS-containing media, media kits were prepared as packaged with FBS and 0.1% phenol red included in the media. For hormone free culture in CSS-containing media, media kits were altered to use CSS in place of FBS and phenol red was omitted. All cells were expanded to passage 3 prior to usage in experiments, and all experiments were preformed using cells from P3 to P6 to minimize variance. Cells were sub-cultured before reaching 80% confluence.

### Estradiol (E2) ELISA

Estradiol (E2) content across lots of FBS and CSS was measured by E2 ELISA (Abcam, ab108667). FBS from 4 suppliers and 5 lots and CSS from 2 suppliers and 3 lots were analyzed for E2 content (Figure S2d). Samples were stored at − 20 °C prior to running the ELISA assay according to manufacturer instructions. Microfluorimetric ELISA data was collected using a SpectraMax i3x plate reader (Molecular Devices). Standard curve analysis was performed to verify assay accuracy (R^2^ = 0.9958, Figure S2b).

### qRT-PCR

HUVEC and HRMVEC from all donors were grown in 6 well plates in the specified media (see Figure captions for details) supplemented with either FBS or CSS. All cells were grown in triplicate with 3 wells per sex per condition per donor. Cells were grown until 80% confluence and collected via trypsinization. Samples were either processed immediately, or stored in RNAlater solution (Invitrogen, AM7020) at 4 °C until isolation. mRNA was isolated from pelleted cells via an RNEasy kit (Qiagen, 74104) according to manufacturer instructions. mRNA quality and concentration were assessed via Nanodrop spectrometry. cDNA was synthesized via an RT^2^ Easy First Strand kit (Qiagen, 330421) according to manufacturer instructions. cDNA was analyzed via a RT^2^ Profiler PCR Array for Human Endothelial Cell Biology (Qiagen, 330231) ran on a StepOnePlus Real-Time PCR system (Applied Biosystems). Data was then exported and analyzed using Qiagen’s GeneGlobe Data Analysis Center.

### Ki67 Immunofluorescent Staining of 2D Cultures

XX and XY HUVEC, HRMVEC, HLF, and HOF were seeded at a density of 10,000 cells per well in 24 well plates and grown in standard media with 2% FBS and 0.1% phenol red, charcoal-stripped media containing 2% CSS, charcoal stripped serum with phenol red containing 2% CSS and 0.1% phenol red, or no serum and only 0.1% phenol red for 48 h. Cells were then washed and fixed in 4% PFA for 15 min at room temperature and washed 2 times in PBS. Monolayers were then blocked and permeabilized via 1% bovine serum albumin (BSA) and 0.1% Triton-X (Sigma Aldrich) in PBS at room temperature for 15 min. Rabbit anti-Ki67 antibody (Abcam, ab16667) was added as supplied without further dilution at 10 ml/ml in 0.1% BSA in PBS on a gentle rock for 2 h at room temperature. Samples were washed with PBS 4 times before incubation in secondary Donkey Anti-Rabbit IgG H&L Alexa Fluor 594 (Abcam, ab150076) at 4 ml/ml, 4 ml/ml 4′,6′-diamidino-2-phenylindole (DAPI) to label nuclei, and 10 ml/ml Alexa Fluor 488 conjugated phalloidin to label actin (Invitrogen, A12379) in 0.1% BSA in PBS for 40 min in the dark with gentle rocking. Plates were then again washed 3 times in PBS and stored covered in PBS at 4 °C protected from light prior to imaging.

### Microphysiological Systems (MPS) Device Fabrication

Detailed fabrication methods used to generate the MPS devices used in this study were reported previously [32]. Briefly, mold designs were drafted in CAD using Fusion 360 (Autodesk) and printed using a Form 3B SLA Printer (Formlabs) in Formlab’s Clear V4 resin (Figure 1c). Molds were then washed in isopropyl alcohol and cured under UV light per manufacturer instructions. To correct warping and to release excess volatiles, molds were then baked at 130 °C clamped between jeweler’s blocks. To produce optically clear PDMS, molds were spray coated with a thin lay of polyurethane coating. PDMS (Sylgard 184, Ellsworth Adhesives) was mixed at a 1:10 ratio of PDMS curing agent to PDMS elastomer by weight and degassed before being poured into prepared molds. PDMS was cured overnight before removing PDMS slabs from the molds. For multilayer endothelial barrier permeability and angiogenesis assay devices, PDMS slabs were then bonded together with a thin PDMS stamp layer. For endothelial barrier permeability devices, the endothelial channel was separated from the stromal compartment and the stromal compartment was separated from the bottom media reservoir using polyester with 0.4 μm pores (Sterlitech). See Figure 3a, b for design details. All PDMS devices were sterilized under UV light for 1 hour. PDMS devices were then functionalized by polydopamine (PDA) coating as previously described and used within 1 week [30, 32].

### Endothelial Barrier FITC-Dextran Permeability Assay and Staining

The endothelial channels of PDA-treated and UV sanitized endothelial barrier devices were incubated with 0.1 mg/ml Geltrex (Gibco, A1413201) at 37 °C for 1 hour to enhance cell adhesion. Geltrex-coated channels were loaded with HUVEC at 8x10^6^ cells/ml and incubated for 1 hour at 37 °C. Unseeded cells were aspirated from the endothelial channel, and VEGM was added to the endothelial channel, the stromal compartment, and both top and bottom media reservoirs. Devices were cultured on a bidirectional rocker housed in a standard incubator at 10 rpm with a 15° angle. This rocking regimen was selected to generate an adequate physiological shear stress (2). After 48 h of endothelial monolayer formation, 2 x 10^6^ HLF/ml embedded in collagen I (2.25 mg/ml) precursor solution were loaded into the stromal compartment. Collagen gels were solidified at 37 °C for 30 min and the bottom media reservoirs were filled with FGM to support fibroblast growth in culture. Media in all compartments was changed daily. Fully loaded devices were cultured in media containing 2% FBS for 48 h at which point a cohort of devices were switched to 2% CSS containing VEGM and FGM. After 24 h, all media was removed from devices and devices were washed with PBS. Media containing 100 μg/ml of 40 kDa FITC-dextran with either 2% FBS or 2% CSS was then introduced into the endothelial channel. The bottom reservoir chamber of the device was loaded with media devoid of FITC-dextran. Devices were returned to culture for 48 h to allow for FITC-dextran transport across the endothelial barrier and through the device layers to lower reservoir. Media in the bottom reservoir was then collected and FITC-dextran fluorescence was measured via a SpectraMax i3x plate reader (Molecular Devices). A standard curve was used to determine the concentration of FITC-dextran in the collected media. Devices cultured in parallel but not utilized for FITC-dextran permeability assays were fixed and stained (Figure 1d).

### Vasculogenesis Assay

PDMS array devices were loaded with collagen I (2.5 mg/ml, Corning) and fibrin (5 mg/ml fibrinogen and 1U/ml thrombin, Sigma Aldrich) co-gels loaded with sex-matched HUVEC and HLF at a density of 2 × 10^6^ cells/ml of each cell type. Hydrogels were polymerized for 15 min at 37 °C before being covered with vascular endothelial growth media (VEGM) (Lifeline Cell Technology) supplemented with 2% FBS and 25 mg/ml aprotinin (EMD Millipore). Cohorts of both XX and XY devices for the 4-, 5-, 6-, and 7-day endpoints were seeded at the same time using the same donor group cells and reagents. For days 5, 6, and 7, devices were further stratified between FBS and CSS groups. 3 gels per sex per time point were analyzed. Tissues within devices were grown in standard culture conditions with media changes occurring at 24 h and every 48 h thereafter. For the first 4 days, all tissues were cultured with VEGM containing 2% FBS. At day 4, the first cohort of tissues were fixed and stained, while the FBS cohorts of days 5, 6, and 7 were kept in FBS supplemented VEGM, and the CSS cohorts were switched to phenol red free VEGM containing 2% CSS. On day 5, tissues from the day 5 FBS and CSS cohorts were fixed, and media changes were performed on the days 6 and 7 cohorts as appropriate. This was repeated on days 6 and 7 (Figure 1e).

### VEGF Gradient-Induced Sprout Angiogenesis Assay

A double lane membrane-free organ chip (MFOC) PDMS device designed for angiogenesis assays was implemented as previously described [30]. Briefly, HLF and HUVEC (2 × 10^6^ cells/ml each) were admixed in 2.2 mg/ml collagen I (Corning), 5 mg/ml fibrinogen (Sigma-Aldrich), and 1 U/ml thrombin (Sigma–Aldrich). The cell-inoculated hydrogel precursor was injected into one of the central tissue compartments of the double MFOC devices and incubated for 15 min at 37 °C. The adjacent central tissue compartment was injected with the same hydrogel mixture without cells and incubated for 15 min at 37 °C. The outer side channels and media reservoirs were filled with VEGF containing VEGM supplemented with 2% FBS and 25 µg/ml aprotinin (EMD Millipore, 616370-20MG). The media was changed after 48 h. On day 3 of culture, the tissues were washed with DPBS twice and the media switched to either FBS or CSS containing VEGM. Additionally, a VEGF-A gradient was set up across the tissues by loading the side channel adjacent to the vascular tissue margin with VEGF-free VEGM and the side channel adjacent to the blank gel is loaded with VEGF containing 10 ng/ml VEGF-A. The corresponding reservoirs were filled with equal volumes of the respective media compositions and VEGF-gradient was maintained every day by replacing half of the culture medium for an additional culture period of 6 days before fixation (Figure 1f).

### 3D Tissue Staining

All tissues were fixed by covering in 4% paraformaldehyde (PFA, Sigma Aldrich), incubated for 1 hour at room temperature, and then overnight at 4 °C. Devices were then washed thoroughly with PBS and stored in PBS at 4 °C until stained. To visualize endothelial cells, devices were stained with 4 µL/mL DAPI to label nuclei, 4 µL/mL Alexa488-conjugated phalloidins to label F-actin, 20 µL/mL DyLight594-conjugated Ulex Europeas agglutinin I (UEA-1, Vector Laboratories) prepared in PBS with 0.2% Triton-X 100 and 1% BSA (Sigma Aldrich) for blocking and permeabilization respectively. Endothelial barrier devices were stained omitting UEA-1. Devices were loaded with the staining cocktail and rocked for 1 hour at room temperature before incubating overnight at 4 °C. After washing, stained devices were protected from light in PBS at 4 °C until imaging.

### Imaging and Image Analysis

All 2D and 3D samples were imaged on an inverted Nikon C2 laser scanning confocal microscope (LCSM) equipped with a Nikon DS-FI3 camera. Samples from a given cohort were imaged at a fixed laser intensity and exposure time to ensure translation between images. 2D samples were imaged at 3 distinct locations in each well to create 3 technical replicates per well. Images were analyzed via ImageJ. Cell counts were determined using the DAPI channel to create a mask and then counting stained nuclei via particle analysis. Similarly, F-actin images were converted to a binary mask for measuring the area of coverage in the image. Ki67-positive cell counts were determined using masks of the Ki67 staining channel and particle analysis as performed to determine total cell counts with DAPI staining. Ki67 indexes for each image were calculated as the percentage of Ki67-positive cells. Monolayers in the channels of endothelial barrier devices were imaged 3 times per device. Images were exported to ImageJ and used to qualitatively assess patency of endothelial barrier formation. For vasculogenesis devices and angiogenesis devices, stained tissues were imaged, and max intensity projection Z-stacks were exported as TIFF files without LUTs. Angiogenesis devices were imaged as a large composite 6x1 stitched image to ensure convergence of morphological variables. Images were then quantified as previously described [32]. All image analysis was completed in MATLAB (R2021b). Briefly, using MATLAB, images were preprocessed, segmented, and analyzed using the open-source segmentation tool REAVER [33]. For quantification of angiogenic sprout growth, differential interference contrast (DIC) images of the central channels of each chip were used to identify the boundary between the blank gel compartment and vascular margin, and morphometric quantification was performed on the regions bound by the central boundary and the ends of the blank gel compartment.

### Statistical Analysis

For RT-qPCR, 4 donors per sex for HUVC and 3 donors per sex for HRMVEC were used, and cells were collected from triplicate wells with n = 3 wells per sex per condition per donor. All RT-qPCR was completed in triplicate for n = 3 technical replicates. Vasculogenesis assays were completed with n = 3 donor pairs per sex with 6 devices per sex per condition. Angiogenesis assays were completed with n = 2 donor pairs per sex with 2-3 replicates per sex for a total of 5 replicates per sex. Per donor averages were calculated prior to analysis. Statistical analyses were performed on aggregated data for each donor using GraphPad Prism V10. Fold changes of cells grown in FBS were compared between genes and conditions via two-way ANOVA with Bonferroni correction. Cell areas, cytoskeletal spreading, and Ki67 positivity were compared across sex and condition via two-way ANOVA. Endothelial permeability was compared between conditions and sexes via two-way ANOVA. Vessel morphometric outputs were compared between sexes and conditions using two-way ANOVA. All statistics were completed at p < 0.05 for significance. Statistical significance definitions are indicated in respective figure captions.

## Results

### Sex-Specific Endothelial Gene Expression Responses to FBS and CSS

XX and XY HRMVEC were grown in either CSS or FBS until 80% confluence and then collected and processed for RT-qPCR analysis (Figure 1a). ELISA was performed to assess E2 levels across multiple lots of FBS and CSS used in this study (Figure S2). Analysis confirmed that mean E2 concentration was significantly higher in FBS (2.7 ± 0.25 nM) compared to CSS (0.20 ± 0.03 nM). Results from a 96 gene endothelial cell-specific qPCR array revealed numerous genes that were differentially expressed between XX and XY endothelial cells exposed to FBS as compared to CSS (Figure 2). XY HUVEC and HRMVEC grown in FBS showed upregulation of genes associated with apoptosis, adhesion, coagulation and thrombosis, and immune and inflammatory responses when compared to CSS cultures (Sex main effect: p < 0.001, Serum main effect: p < 0.001, Figure 2b, e, f, g). Conversely, XX HUVEC and HRMVEC downregulated genes in these functional groups in FBS as compared to CSS (Sex main effect: p < 0.001, Serum main effect: p < 0.001, Figure 2b, e, f, g). XY HUVEC and HRMVEC in FBS significantly upregulated genes associated with vasoconstriction, including *AGT* (HRMVEC p = 0.006, HUVEC p = 0.032), and *AGTR1* (HRMVEC p < 0.001, HUVEC p < 0.001), while simultaneously significantly downregulating genes associated with vasodilation, including *NOS3* (HRMVEC p = 0.048, HUVEC p = 0.09 (ns)) and *NPR1* (HRMVEC p < 0.001, HUVEC p = 0.049) (Figure 2a). XX cells showed opposite expression patterns of these genes, highlighting consistent sex difference in the gene expression response to FBS (Sex main effect: p < 0.001, Serum main effect: p < 0.001, Figure 2a). Additionally, XX cells upregulated genes associated with ECM production, with *COL18A1* (HRMVEC p = 0.038, HUVEC p = 0.046) being an exception to this trend (Figure 2d). Of note, fold changes of XX and XY cells grown in FBS were significantly different between the sexes in many of the genes analyzed (Figure S3). Taken together, these gene expression patterns suggest that FBS induced XX cells to upregulate genes reflective of normal vascular function and caused XY cells to upregulate genes that have been associated with vascular dysfunction.

### Sex-Specific Effects of FBS and CSS on Cell Proliferation and Morphology in 2D Culture

Cell proliferation in expansion cultures is a critical step to generate the required numbers of primary cells for tissue engineering applications [34]. We assessed cell morphology and metrics of proliferation for both XX and XY endothelial cells and fibroblasts cultured in FBS- and CSS-containing media for 48 h via staining and image analysis (Figure 1b). Gross cell counts per image via nuclear staining were used to enumerate cell numbers (Figure 3a, b, d), cell cycle participation was assessed via immunofluorescent staining for Ki67 (Figure 3a, b, e), and cytoskeletal coverage as a percentage of image area as shown via F-actin staining was used as a morphological readout (Figure 3a, b, c). To verify that measured effects of switching from FBS to CSS were not due to the absence of phenol red in CSS, we assessed these metrics first in cells grown in FBS, CSS, CSS with added phenol red, or serum-free medium with added phenol red. There were no significant effects on cell counts, cytoskeletal areas, or Ki67 indexes in any of the cell types tested when comparing CSS only and CSS with phenol red (Figure S4).

F-actin staining showed increased cytoskeletal coverage in XY HUVEC (28 + /− 3.1%), HLF (28.4 ± 2.2), and HOF (27.4 ± 1.1%) grown in CSS compared to cells grown in FBS (HUVEC: 17.8 ± 0.3%, p = 0.04; HLF: 16 ± 2.3%, p < 0.001; HOF: 14 ± 0.1 %, p = 0.008; Cytoskeletal area sex main effect = p < 0.001, cytoskeletal area serum main effect p = 0.55, cytoskeletal area interaction effect = p < 0.001). XY HRMVEC did not exhibit a significant difference between FBS (34.7 ± 2.1%) and CSS (30.5 ± 2.6%, p = 0.78). Conversely, XX HLF and HOF showed significantly increased areas in FBS (HLF: 28 ± 1.3%, HOF: 30 ± 3.4%) compared to CSS (HLF: 16.4 ± 1.1%, p = 0.006; HOF: 16.7 ± 2.3%, p < 0.001). XX HUVEC followed the same trend with greater area in FBS (27.7 ± 0.6%) than in CSS (20.9 ± 1.3%, p = 0.06), and no significant difference in area was seen in XX HRMVEC (CSS: 29.5 ± 1.1%, FBS: 28 ± 1.3%, p = 0.90) or HUVEC (Figure 3a, b, c). Regarding sex differences within a serum condition, XX cells generally exhibited higher Ki67 indexes and cell areas compared to XY cells when grown in FBS, but these metrics were higher in XY cells when cultured in CSS (Figure S5). Cell area differences batch measured in several cells per image were corroborated using high magnification images to measure single-cell cytosolic area (Figure S6).

There were no significant differences in nuclei counts between conditions in any of the 4 cell types tested in either sex (Cell count sex main effect = p < 0.001, cell count serum main effect = p = 0.31, cell count interaction effect = p = 0.41. Figure 3d), but Ki67 indexes revealed significant differences in cell cycle status (Ki67 sex main effect = p < 0.001, Ki67 serum main effect = p = 0.51, Ki67 interaction effect = p < 0.001. Figure 3e). In XY HUVEC, HRMVEC and HOF, the percentage of cells positive for Ki67 was significantly greater in cells grown in CSS (HUVEC: 39.7 ± 2.5%, HRMVEC: 65.4 ± 9.2%, HOF: 56.7 ± 6.3%) as compared to cells grown in FBS (HUVEC: 12.8 ± 1.9%, p < 0.001; HRMVEC: 29.9 ± 4.7%, p < 0.001; HOF: 30.5 ± 1.2%, p < 0.001). XY HLF, while not significantly different, also followed this trend (CSS: 26.5 ± 0.8%, FBS: 21.8 ± 0.5%, p > 0.99). Conversely, XX HUVEC and HRMVEC showed greater Ki67 positivity in FBS (HUVEC: 60 ± 2.6%, HRMVEC: 36.6 ± 3.7%) than in CSS (HUVEC: 18.9 ± 0.5%, p < 0.001; HRMVEC: 14.4 ± 2.2%, p = 0.003). No significant difference is seen in XX HLF (FBS: 29.2 ± 1%, CSS: 16.1 ± 2.4%, p = 0.14) and HOF (FBS: 40.6 ± 1%, CSS: 41.6 ± 2.1%, p > 0.99). It is important to note that Ki67 is expressed in cells in all stages of the cellular proliferation [35], so while cell counts are not significantly different at 48 h, cells may show differences in cell counts at later time points based on differences in cell cycle participation.

### Sex-Specific Effects of FBS and CSS on Endothelial Barrier Integrity and Permeability

A 3D MPS model of a single vessel endothelial barrier was used to assess sex-specific effects of FBS and CSS on barrier patency and macromolecular permeability (Figure 1d, see Materials and Methods for details). After 4 days of establishing endothelial barriers in FBS-containing medium, devices were cultured with FBS- or CSS-containing medium for an additional 48 h. FITC-dextran was added to the media perfused through the endothelial channel to assess barrier permeability. In parallel, other devices were fixed and stained for F-actin and DAPI to assess endothelial barrier organization and channel coverage (Figure 1d, 4a–c). Qualitatively, XX HUVEC cultured with FBS displayed cytocortical F-actin localization at cell boundaries and complete channel coverage reflective of barrier formation, while channel coverage was decreased in XX devices cultured with CSS. XY devices, conversely, displayed incomplete channel coverage in FBS and improved channel coverage in CSS (Figure 4d). Sex-specific effects of FBS and CSS on endothelial barrier morphology translated to marked differences in macromolecular permeability. Overall, XX devices displayed lower transferred concentrations of 40 kDa FITC-dextran than XY devices (Permeability sex main effect = p = 0.002, permeability serum main effect = p = 0.37, permeability interaction effect = p = 0.02). Within XX devices, FBS resulted in an average transferred FITC-dextran concentration of 1.547 ± 0.488 ng/ml in the collection channel, whereas collected medium from XX devices cultured in CSS had an average transferred FITC-dextran concentration of 3.196 ± 0.565 ng/ml (p = 0.05). Permeability in XY devices was increased by FBS compared to CSS (FBS = 7.428 ±0.593 ng/ml, CSS = 4.310 ±0.627, p < 0.001) (Figure 4e). Taken together, these data suggest that FBS enhances endothelial barrier function in XX devices, while switching to CSS enhances barrier function in XY devices.

### Sex-Specific Effects of FBS and CSS on Bulk Tissue Vasculogenesis

Facilitating robust vasculogenesis is often a critical component of engineering vascularized tissue constructs and MPS. Sex-specific effects of FBS and CSS on bulk tissue vasculogenesis were assessed using a simple PDMS culture device to form 3D tissues comprised of combinations of HUVEC and HLF embedded in a blended collagen type I and fibrin hydrogel [36, 37] (Figure 1e). Tissues were cultured in FBS-containing media for 4 days to facilitate cell adhesion and integration in the 3D tissue environment before switching half of the tissue constructs to CSS-containing media to measure the effect of removing FBS on vascular network maturation. We quantified the relative effects of FBS and CSS on vasculogenesis within each sex (Figure 5), and the impact of cell sex on vasculogenesis for each serum condition (Figure S7).

XX tissues qualitatively formed more robust networks compared to XY tissues after the initial 4 days of culture in FBS (Figure 5a), although there were no statistically significant sex differences in vessel density, total vessel length, branchpoint count, or vessel area fraction at this time point (Figure S7). XX networks maintained in FBS up to 7 days continued to show signs of maturation with significantly increased metrics of vasculogenesis (Figure 5b–e). Conversely, XX vessel networks grown in CSS began to show signs of regression in days 5 through 7 (Figure 5a), as evidenced by decreased metrics of vasculogenesis relative to maintenance in FBS at day 7 (Figure 5b–e; see Figure 5 caption for two-way ANOVA analysis of overall sex and serum effects). Markedly different trends were measured in XY tissues. XY tissues that were switched to CSS at day 4 showed increased network formation relative to FBS cultures by day 7 (Figure 5b–e). XY vascular networks maintained in FBS showed significantly lowered vessel density, total length, branchpoint count, and area fraction when compared to XX devices in FBS at days 5 through 7 (Figure S7). Switching XY devices to CSS at day 4 resulted in significantly increased vessel area fraction, branchpoint count, and vessel density by day 7 (Figure S7a, c, d). Taken together, these data suggest that FBS preferentially enhances XX tissue vasculogenesis, while switching to the hormone-free milieu of CSS enhances XY tissue vasculogenesis.

### Sex-Specific effects of FBS and CSS on Sprouting Angiogenesis

Vascular development typically occurs via a combination of vasculogenesis that establishes tubular networks and sprouting angiogenesis that refines network architecture. While vasculogenesis is relevant in the context of developmental biology and tissue construct assembly, sprouting angiogenesis is often studied in the contexts of adult tissue physiology and disease pathophysiology [38–40]. We utilized a membrane free organ chip that enables the formation of two adjacent and contiguous tissue layers patterned via surface tension as reported elsewhere [30]. One of the tissue layers was seeded with sex-matched HUVEC and HLF in a collagen type I and fibrin hydrogel blend, and the adjacent tissue layer was loaded with an acellular hydrogel of the same composition. Devices were cultured for 4 days in FBS before applying a VEGF-A gradient to induce directional sprouting into the acellular tissue layer. One cohort of devices within each sex were then switched to CSS-containing media while the remainder were maintained in FBS. All devices were cultured to a total of 9 days (Figure 1f).

Sprouting angiogenesis was significantly enhanced in XX devices when compared to XY devices in both FBS and CSS conditions (Figure 6a; see Figure 6 caption for two-way ANOVA analysis of overall sex and serum effects). XX devices in FBS had a significantly increased sprout density (p < 0.001), sprout area fraction (p < 0.001), and sprout length (p < 0.001) when compared to XY devices in FBS (Figure 6b–d). Similarly, in CSS, XX devices maintained an increased sprout density (38 ± 10 mm/mm^2^ in CSS compared to 47 ± 5 mm/mm^2^ in FBS, p = 0.36), total sprout length (8519 ± 2910 μm in CSS, 16142 ± 2788 μm in FBS, p = 0.02), and sprout area fraction (0.08 ± 0.02 in CSS compared to 0.13 ± 0.01 in FBS, p = 0.02) (Figure 6b, c). Collectively, these data suggest that XX devices exhibited enhanced overall angiogenic potential, but this sex difference is more pronounced in FBS than in CSS.

Regarding the impact of serum type within each sex, we measured significantly increased sprout area fraction (p = 0.02) and sprout length (p = 0.02) in FBS devices compared to CSS devices (Figure 6c, d). By contrast, there were no significant differences between FBS and CSS in XY devices for any of the metrics analyzed (Figure 6b–d). In contrast to results of the MPS vasculogenesis assay (Figure 5), analysis of initial vascular network formation in the fully vascularized tissue layer of the angiogenesis MPS devices revealed no significant differences between FBS and CSS in XY devices (Figure S8b–e). In line with results from the vasculogenesis model, there were trends of enhanced XX network assembly in FBS relative to CSS, but only branchpoint counts were significantly different (Figure S8d). These data demonstrate a significant sex difference in the angiogenic response to externally applied VEGF-A gradients in the presence of FBS and highlight the interplay between media composition and specific MPS format.

## Discussion

### FBS Imparts Sex-Specific Effects in 2D Cell Culture and MPS

As MPS and other tissue-engineered models are increasingly adopted as preclinical tools, sex-specific *in vitro* culture methods must be developed to improve biomimicry and align with NIH directives [25, 41]. Accordingly, *in vitro* systems should be developed with careful consideration of how components of the culture environment, including hormonally variable culture medium supplements like FBS, may exert sex-specific effects. Currently, there are no standardized sex-specific cell culture protocols that account for the influence of sex hormones on cellular physiology. Focusing on sex differences in the response to common media formulations, we demonstrated significant sex-specific effects of FBS in a range of 2D and 3D culture models. Notably, female (XX) cells exhibit enhanced cytoskeletal spreading, proliferation, and upregulation of genes related to endothelial cell function when cultured in FBS (Figures 2, 3, S3, S5, S6). Conversely, male (XY) cells cultured with FBS exhibit suppressed proliferation and downregulation of the same endothelial genes (Figures 2, 3, S3, S5, S6).

Despite the widespread use of FBS in human cell culture applications for numerous decades, potential sex-specific effects of FBS in human cell culture systems and their impact on assay readouts have largely been overlooked and remain poorly investigated. A limited number of previous studies have highlighted the influence of sex hormones present in FBS on the plating and growth of human tumor cell lines and animal cells [42–44]. While our study clearly demonstrates sex-specific effects of FBS on primary human cells, the underlying mechanisms are unknown. E2 ELISA data suggests that enhanced XX cell performance across assays in the presence of FBS may be due to the sex-specific actions of E2 (Figure S2). The average E2 concentration across the tested lots of FBS aligns with previously reported values (Figure S2c) [45]. Importantly, while the average E2 level was within the physiological range for post-pubescent and pre-menopausal female patients, this level is significantly higher than the average range of E2 in males [46, 47]. Accordingly, it may be appropriate to think of FBS-containing medium as a female hormonal environment, due to the high level of E2 and relatively low levels of androgens such as testosterone [45].

The high variability of FBS between suppliers and lots can be influenced by factors that are difficult to control including the climate where the FBS was harvested [23]. Suppliers do not provide information regarding hormone levels in FBS, so repeat testing on a per lot basis would be required to track hormone levels in culture. To counteract this variability, FBS may be charcoal stripped to produce hormone-depleted serum, herein referred to as CSS [24]. However, charcoal-stripping also depletes other sex hormones such as androgens and important growth factors such as insulin that may modulate cell growth in culture [24, 48]. Our study demonstrates that using hormone-depleted CSS mitigates the inhibitory effects of FBS in XY culture models and MPS (Figures 2, 3, 4, 5, 6), but still exerts sex-specific effects evidenced by the lasting differences in endothelial permeability, vasculogenesis, and angiogenesis between XX and XY devices cultured with CSS (Figures 4, 5, 6, S7, S8). Hence, while FBS induces a sex-specific response in culture, the hormone-depleted milieu of CSS-containing medium does not produce sex-equivalent responses in the range of 2D assays and MPS used in this study. The persistent sex difference in response to CSS may be due to aforementioned factors including the lack of sex-specific hormone signaling or nutrient deprivation due to charcoal stripping.

Serum-free media formulations devoid of exogenous sex hormones are commonly used for various cell culture and tissue engineering applications [49]. For some applications, such as tissue-engineered meats, the removal of animal-based serum products is desired to ensure food safety and maintain ethical integrity [50]. Physiological differences between animal and human serums are also believed to be a confounding variable drug development studies [51]. More specifically, serum-free formulations have been developed for cell types such as neurons in which FBS can impart negative effects such as decreased synaptic signaling and decreased neuronal differentiation [52–54], but the sex-specificity of these effects have not been investigated. Engineering sex-specific formulations of serum-free media will benefit from studies that define the sex-specific effects of the complex mixture of growth factors, nutrients, hormones, and other vital components naturally present in FBS [50, 55].

Herein, we began by expanding cells in FBS before switching to CSS in the employed assays. While our long-term goal is to eliminate the use of FBS and develop fully sex-specific protocols for expansion culture and MPS assays, it is important to note that many commercially available primary cells are supplied in complete growth media supplemented with FBS [56]. Additionally, many researchers utilize FBS in cell seeding protocols due to the effects of components such as fibronectin and laminin that promote cell adhesion and spreading [57]. Therefore, understanding the sex-specific effects of FBS, and of removing FBS-derived components that are depleted by charcoal stripping, is relevant to guiding the interpretation of much current and past research on the use of human cell culture technologies.

### Sex-Specificity in MPS Models of the Vasculature

Sex differences in the proliferation and cell spreading responses to FBS translated into differences in endothelial permeability, vasculogenesis, and angiogenesis (Figures 4, 5, 6, S7, S8). Increased patency and decreased permeability of XX endothelial barriers (Figure 4) and enhanced vasculogenesis in XX tissues (Figure 5) relative to the XY counterparts grown in FBS supports the hypothesis that estrogenic signaling from FBS is beneficial to XX cells, although experiments testing targeted inhibition of estrogen signaling in FBS cultures would be required to con. Many clinical studies strongly suggest that E2 promotes vascular health and protects against cardiovascular disease in female patients [58–60]. Cardiovascular health risks remain low in female patients prior to menopause but increase during menopause as E2 levels drop [61]. Upregulation of genes promoting increased vascular health by XX cells in our experiments is congruent with these clinical observations (Figure 2, S3). Sex-specific MPS models of the vasculature could provide clinically relevant venues for investigating the impact of varying levels of E2 and other sex hormones in the context of hormone replacement therapies and other treatments based on pharmacological modulation of sex hormone signaling.

Our study demonstrates that the inhibitory effects of FBS, and the associated imbalance of estrogenic and androgenic signals, on XY cells are at least partially reversible by switching to the hormone-depleted milieu imparted by CSS. The sex-specific inhibitory effects of FBS on XY vascular networks were partially reversed when cultured in CSS for 3 days in the MPS model of bulk vasculogenesis (Figure 5). Similarly, the switch to CSS significantly improved XY endothelial barrier function compared to maintenance in FBS (Figure 4). On the other hand, XY cells in angiogenesis models failed to exhibit substantial sprouting in either FBS or CSS (Figure 6). Angiogenesis and vasculogenesis are different morphogenetic processes regulated by distinct and context-dependent signaling pathways and associated patterns of gene expression [62]. The contrasting results from our MPS models of vasculogenesis and angiogenesis support the notion that hormonal environments may differentially alter these processes. While the literature suggests that there are important sex-specific effects of estrogens and testosterone in angiogenesis, less is known about the role of sex hormones in vasculogenesis [63–66]. Engineering sex-specific MPS models of the vasculature can simultaneously provide venues for deeper mechanistic investigation of sex hormone effects on different aspects of vascular physiology and improve the translational relevance of MPS for applications in pharmacology and therapeutic screening.

### Implications for Cell Culture and MPS in Preclinical Research

Cells utilized within culture systems range from immortalized cell lines to primary cells, each offering unique advantages and limitations in studying cellular physiology. While cell lines often provide a reproducible and renewable cell source, hormone receptors may be lost due to genetic drift over prolonged passages [67, 68]. In contrast, primary cells isolated directly from human tissues retain native hormone receptor profiles which better reflect physiological conditions [5, 69, 70]. Importantly, primary human cells are collected from donors with *in vivo* sex-specific hormonal axes that impart baseline phenotypic differences between male and female cells. Understanding the impact of varying hormone levels between the sexes and at different stages of life are foundational aspects of endocrinology and sex-based biology research and should be considered when developing and implementing human cell-based culture models and MPS. Improving the reproducibility of *in vitro* cell culture systems such as MPS is an ongoing challenge [23, 71]. Improving physiological relevance of MPS by incorporating an internal vasculature is a central goal of the field [72]. Accounting for sex-specific hormone effects on vascular physiology and pathophysiology will improve both accuracy and reproducibility in vascularized MPS models of diseases including diabetes and cancer [73–75]. Minimizing reliance on inherently variable components such as FBS, or at least accounting for their sex-specific effects, will help achieve a higher degree of reproducibility. Further, other components of cell culture, such as cell culture plastics and phenol red that can mimic sex hormones and potentially impart sex-specific effects that influence assay readouts should be considered [76]. While we did not detect any significant effects of phenol red in the assays used for this study (Figure S4), phenol red at concentrations used in some media formulations has been shown to induce estrogenic signaling in multiple cell types [20, 77].

### Future Directions

While our study focuses on endothelial cells and fibroblasts, the effects of FBS and CSS on other cell types must be explored to broaden the scope and relevance of this work. Of note, we saw similar responses between two different types of endothelial cells (HUVEC and HRMVEC) and two types of fibroblasts (HOF and HLF), with largely conserved effects in cells derived from multiple tissues. While our study suggests that results may translate across tissue types, expanded studies should examine more cell types such as epithelial cells and neurons to address cell type-specific sex differences. Additionally, CSS did not establish an equivalent baseline between female and male cells in our cell culture and MPS assays, thus further development of serum-free or hormone-defined media that can mimic biological environments is necessary. We have previously reported the sex-specific effects of defined concentrations of exogenous E2 and dihydrotestosterone (DHT) added to CSS-containing medium on bioenergetic capacity and vasculogenesis [29]. Ongoing work should focus on the development and testing of media formulations with more complex combinations of estrogens, androgens, and other sex hormones such as progesterone. It will be imperative to establish benchmarks of comparison to define sex-specificity in cell culture systems and MPS. Ultimately, the translational relevance of sex-specific MPS will need to be validated through recapitulation of clinically documented sex differences in the manifestation and treatment responses of various diseases.

## Supplementary Information

Below is the link to the electronic supplementary material.

Supplementary file1 (PDF 4282 kb)

## Data Availability

Data is available at 10.6084/m9.figshare.29950229.v1. Any other data and more detailed explanations of laboratory procedures will be made available upon reasonable request to the corresponding author.

## Abbreviations

NIH: National Institutes of Health
FBS: Fetal bovine serum
CSS: Charcoal stripped serum
FDA: Federal Drug Administration
MPS: Microphysiological systems
HUVEC: Human umbilical vein endothelial cells
HLF: Human lung fibroblasts
HRMVEC: Human microvascular endothelial cells
HOF: Human ocular fibroblasts
