# Correction: Fetal Bovine Serum Modulates Primary Human Cell Phenotypes, Endothelial Barrier Function, Vasculogenesis, and Angiogenesis in a Sex-Specific Manner

**DOI:** 10.1007/s12195-025-00871-0

**Published:** 2025-09-24

**Authors:** Ashley Martier, G. Wills Kpeli, Keefer Boone, Isabella R. Posey, Mark J. Mondrinos

**Affiliations:** 1 Department of Biomedical Engineering, Tulane University School of Science & Engineering, 6823 St. Charles Avenue, New Orleans, LA 70118 USA; 2 Tulane Center of Excellence in Sex-based Precision Medicine, New Orleans, LA USA; 3 Department of Physiology, Tulane University School of Medicine, New Orleans, LA USA; 4 Tulane Cancer Center, Louisiana Cancer Research Center, New Orleans, LA USA

**Correction to: Cellular and Molecular Bioengineering** 10.1007/s12195-025-00860-3

The original article has been updated to add the missing Electronic Supplemental Material.

