# Supplementary material for: Fetal Bovine Serum Modulates Primary Human Cell Phenotypes, Endothelial Barrier Function, Vasculogenesis, and Angiogenesis in a Sex-Specific Manner
Source: Cell Mol Bioeng. 2025 Sep 5;18(5):433–49. doi: 10.1007/s12195-025-00860-3 (PMC12579606; doi:10.1007/s12195-025-00860-3)
Supplement: Supplementary file 1 — Supplementary file1 (PDF 4282 kb) [file 12195_2025_860_MOESM1_ESM.pdf]

Supporting Information for:

Fetal bovine serum modulates primary human cell phenotypes, endothelial barrier function, vasculogenesis, and angiogenesis in a sex-specific manner

Ashley Martier[1], G. Wills Kpeli[1], Keefer Boone[1], Isabella R. Posey[1], Mark J. Mondrinos[1, 2, 3,*]

1 – Department of Biomedical Engineering, Tulane University School of Science & Engineering, New Orleans, LA, USA
2 – Tulane Center of Excellence in Sex-based Precision Medicine, New Orleans, LA, USA
3 – Department of Physiology, Tulane University School of Medicine, New Orleans, LA, USA
4 – Tulane Cancer Center, Louisiana Cancer Research Center, New Orleans, LA, USA

* Correspondence should be addressed to:
Mark J. Mondrinos, Ph.D.
mmondrinos@tulane.edu

List of Contents

Supplemental Figures

Figure S1 – Cell donor information.
Figure S2 – ELISA analysis of E2 levels in multiple lots of FBS and CSS from various suppliers.
Figure S3 – RT-qPCR analysis of sex-specific effects of FBS and CSS.
Figure S4 – Phenol red does not significantly alter the sex-specific effects of FBS and CSS on cytoskeletal spreading and Ki67 indexes.
Figure S5 – Sex- and serum-specific effects on cytoskeletal areas and Ki67 indexes.
Figure S6 – Sex-specific effects of FBS and CSS on cell area.
Figure S7 – Sex-specific effects of FBS and CSS in the bulk vasculogenesis MPS model.
Figure S8 – Sex-specific effects of FBS and CSS on initial vascular network assembly in the MPS angiogenesis model.

Cell donor information

Human Umbilical Vein Endothelial Cells (HUVEC)

Supplier	Catalog #	Donor #	Donor Sex	Donor Race	Donor Age
Lonza	CC-2517	0000315132	Female	Caucasian	N/A
Lonza	CC-2517	0000234484	Female	Caucasian	N/A
Stemcell	200-0630	W243422007450P	Female	N/A	N/A
ATCC	PCS-100-010	70013502	Female	N/A	N/A
Lonza	CC-2517	0000318930	Male	Caucasian	N/A
Lonza	CC-2517	0000273864	Male	Caucasian	N/A
Stemcell	200-0630	W243422007461J	Male	N/A	N/A
Stemcell	200-0630	W243422007038J	Male	N/A	N/A

Human Lung Fibroblasts (HLF)

Supplier	Catalog #	Donor #	Donor Sex	Donor Race	Donor Age
ATCC	PCS-201-013	70014625	Female	Caucasian	29
ATCC	PCS-201-013	700451445	Female	Caucasian	20
Lonza	CC-2512	22TL151174	Female	Caucasian	45
Lonza	PCS-201-013	343490	Female	Caucasian	24
ATCC	PCS-201-013	70002722	Male	Hispanic	14
Lonza	CC-2512	23TL268440	Male	Hispanic	45
Lonza	CC-2512	23TL275471	Male	Black	32
Lonza	CC-2512	0000615568	Male	Hispanic	22

Human Retinal Microvascular Endothelial Cells (HRMVEC)

Supplier	Catalog #	Donor #	Donor Sex	Donor Race	Donor Age
Celprogen	36052-03	230501	Female	Caucasian	35
Celprogen	36052-03	230371	Female	Asian	25
Celprogen	36052-03	230625	Female	Caucasian	30
Celprogen	36052-03	230502	Male	Caucasian	37
Celprogen	36052-03	230002	Male	Black	41
Celprogen	36052-03	230666	Male	Caucasian	28

Human Ocular Fibroblasts (HOF)

Supplier	Catalog #	Donor #	Donor Sex	Donor Race	Donor Age
CellBiologics	H-6204	208536	Female	Caucasian	36
ScienCell	6620	30574	Female	Caucasian	26
ScienCell	6620	30448	Female	Black	39
CellBiologics	H-6204	208206	Male	Caucasian	40
ScienCell	6620	30733	Male	Hispanic	32
ScienCell	6620	30222	Male	Caucasian	17

Figure S1: Cell donor information. Cells used in this study with supplier, supplier catalog number, donor number, and donor characteristics. Cells were used throughout as noted in methods sections and figure captions.

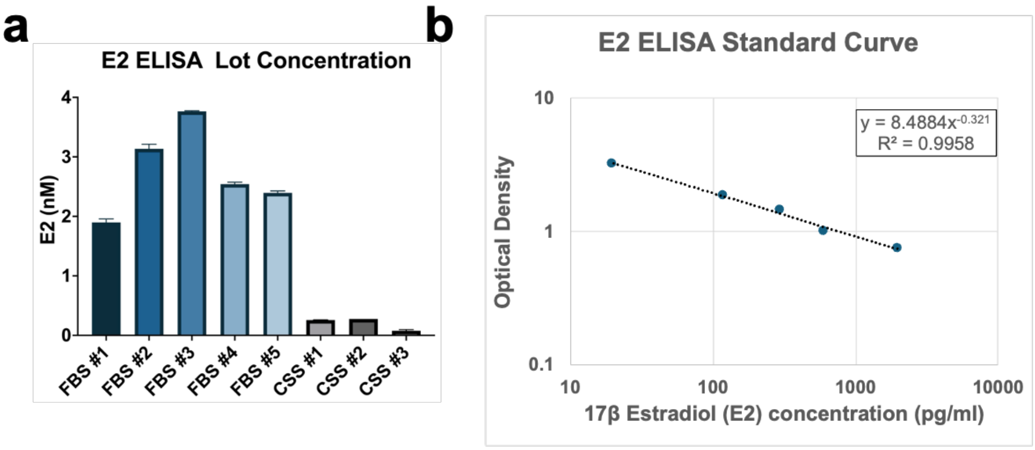

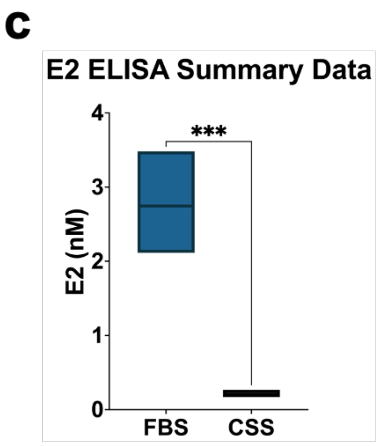

d

Sample	Supplier	Catalog #	Lot
FBS #1	Lifeline Cell Technologies	LS-1012	11553
FBS #2	Lifeline Cell Technologies	LS-1012	11269
FBS #3	ATCC	30-2020	3005151
FBS #4	Gibco	A56707-01	C2930772RP
FBS #5	Corning	35-015-CV	07825004
CSS #1	Gibco	12676029	76-029
CSS #2	Gibco	12676029	74-229
CSS #3	Corning	35-072-CV	02524002

Figure S2: ELISA analysis of E2 levels in multiple lots of FBS and CSS from various suppliers. a: E2 was elevated in all FBS lots tested compared to CSS lots. Error bars represent SEM. **b:** Concentrations of E2 were quantified using a standard curve constructed with the manufacturer provided E2 standards. Equation for best fit and R^2 displayed on graph. **c:** The average level of E2 in FBS was 2.7 +/- 0.25 nM while the average across CSS was 0.2 +/- 0.03 nM. FBS E2 levels were significantly increased compared to CSS (p=0.004). Compared via unpaired t-test with Welch's correction in Graphpad Prism. **d:** Supplier information for FBS and CSS samples tested.

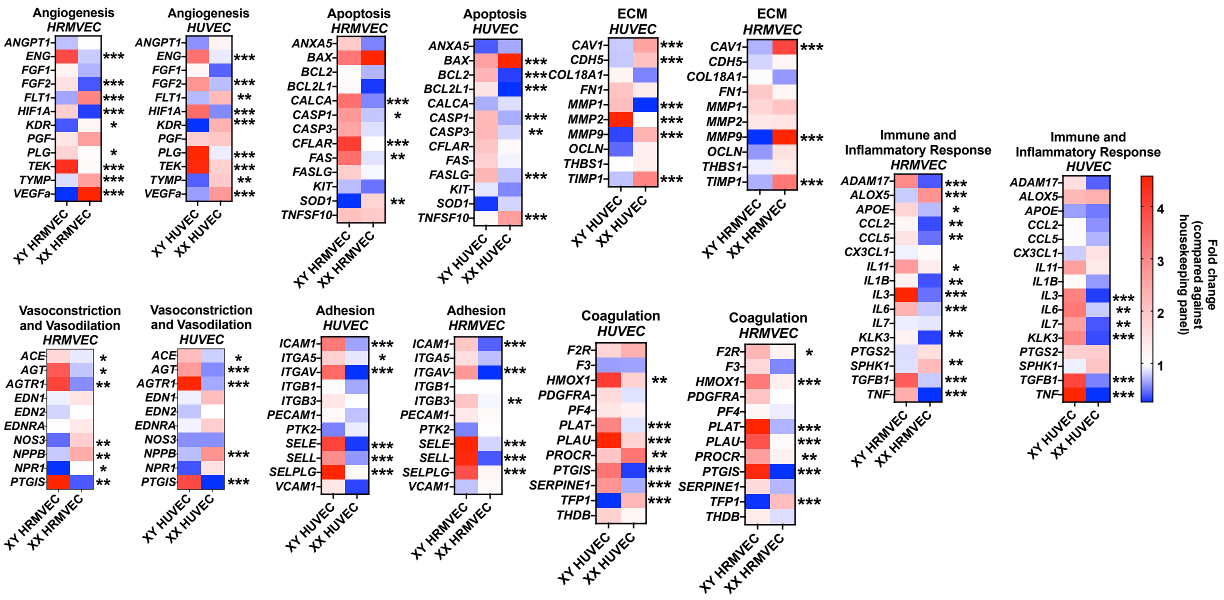

Figure S3: RT-qPCR analysis of sex-specific effects of FBS and CSS. HUVEC and HRMVEC grown in CSS and FBS containing media showed significant differences between sex in endothelial functional genes quantified from Qiagen Endothelial Gene arrays. White cells represent a fold change of 1 (i.e. no change between FBS and CSS) with blue showing downregulation and red showing upregulation. Compared to housekeeping panel (standardized to fold change = 1) in same cells grown in CSS via two-way ANOVA with Bonferroni correction. * = $p < 0.05$, ** = $p < 0.01$, *** = $p < 0.001$. n=3 donors per sex for HRMVEC, 4 donors per sex for HUVEC; 3 technical replicates per donor. Asterisks next to rows signify significance in the genes immediately to the left between XX and XY groups.

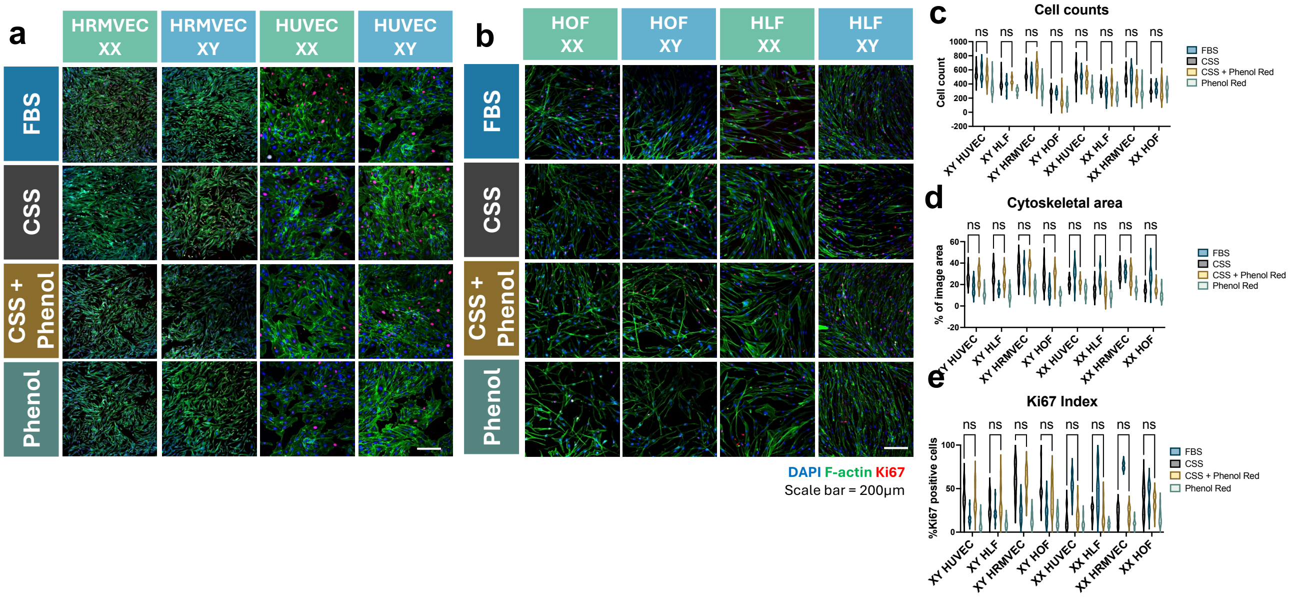

Figure S4: Phenol red does not significantly alter the sex-specific effects of FBS and CSS on cytoskeletal spreading and Ki67 indexes. a: Representative micrographs of XX and XY HUVEC and HRMVEC grown in FBS, CSS, CSS + phenol red, or only phenol red for 48 hours stained for DAPI (blue), F-actin (green), and Ki67 (red). Scale bar = 200μm. **b**: Representative micrographs of XX and XY HLF and HOF grown in FBS, CSS, CSS + phenol red, or only phenol red for 48 hours stained for DAPI (blue), F-actin (green), and Ki67 (red). Scale bar = 200μm. **c**: Cytoskeletal area was not significantly different between CSS and CSS + phenol red groups in either sex. **d**: Cell count was not significantly different between media conditions in either sex or any cell type. **e**: Ki67 positivity remained unchanged in the presence of phenol red compared to CSS alone. * = $p < 0.05$, ** = $p < 0.01$, *** = $p < 0.001$. All statistics were analyzed via two-way ANOVA in GraphPad Prism. Error bars represent SEM. n=3 donors per sex for HOF, HRMVEC; 4 donors per sex for HLF, HUVEC. 3 wells per donor, 3 images per well.

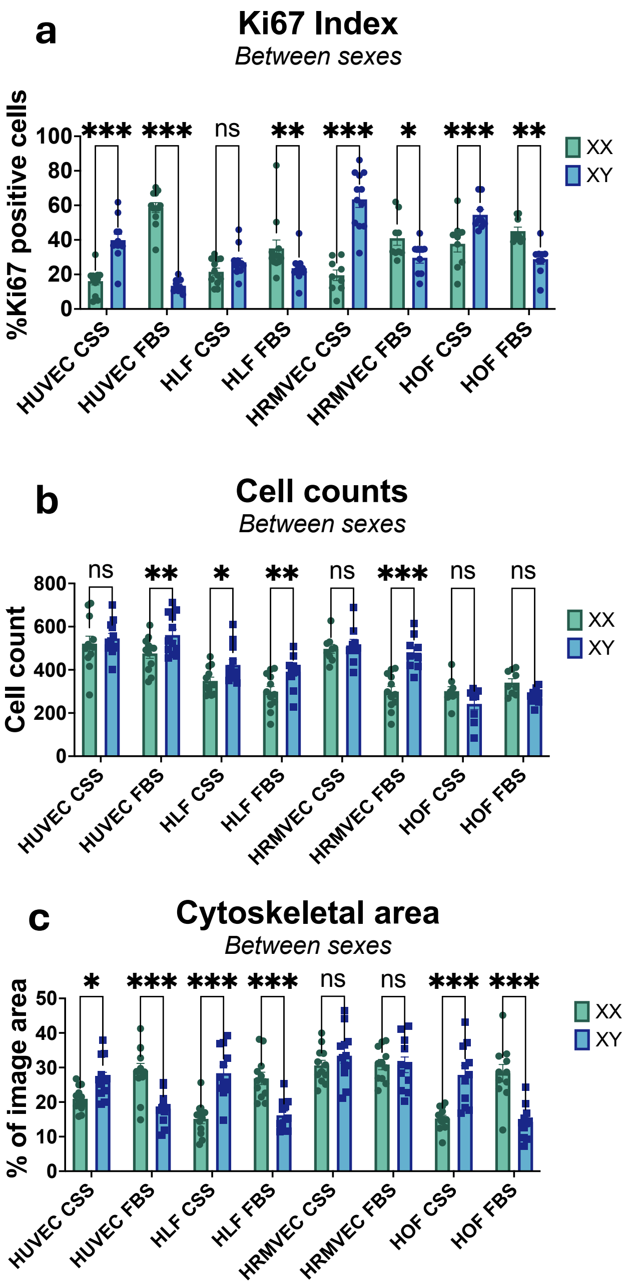

Figure S5: Sex- and serum-specific effects on cytoskeletal areas and Ki67 indexes. a: Cytoskeletal area was significantly greater in XX HUVEC, HLF, and HOF in FBS when compared to XY HUVEC, HLF and HOF in CSS. Conversely, XY HLF and HOF cell areas were significantly greater than XX HLF and HOF cell areas in CSS. No significant difference was noted between XX and XY HUVEC in CSS or XX and XY HRMVEC in either FBS or CSS. **b:** XY HLF cell counts were significantly greater in FBS compared to XX HLF, but no other significant difference in cell counts were noted between sexes. **c:** XX HUVEC Ki67 indexes were significantly higher than XY HUVEC in FBS. XY HUVEC and HRMVEC Ki67 indexes in CSS were significantly higher compared to XX cells. * = $p < 0.05$, ** = $p < 0.01$, *** = $p < 0.001$. All statistics were analyzed via two-way ANOVA in GraphPad Prism. Error bars represent SEM. n=3 donors per sex for HOF and HRMVEC. 4 donors per sex for HLF and HUVEC. 3 wells per donor, 3 images per well. Dots represent per well average.

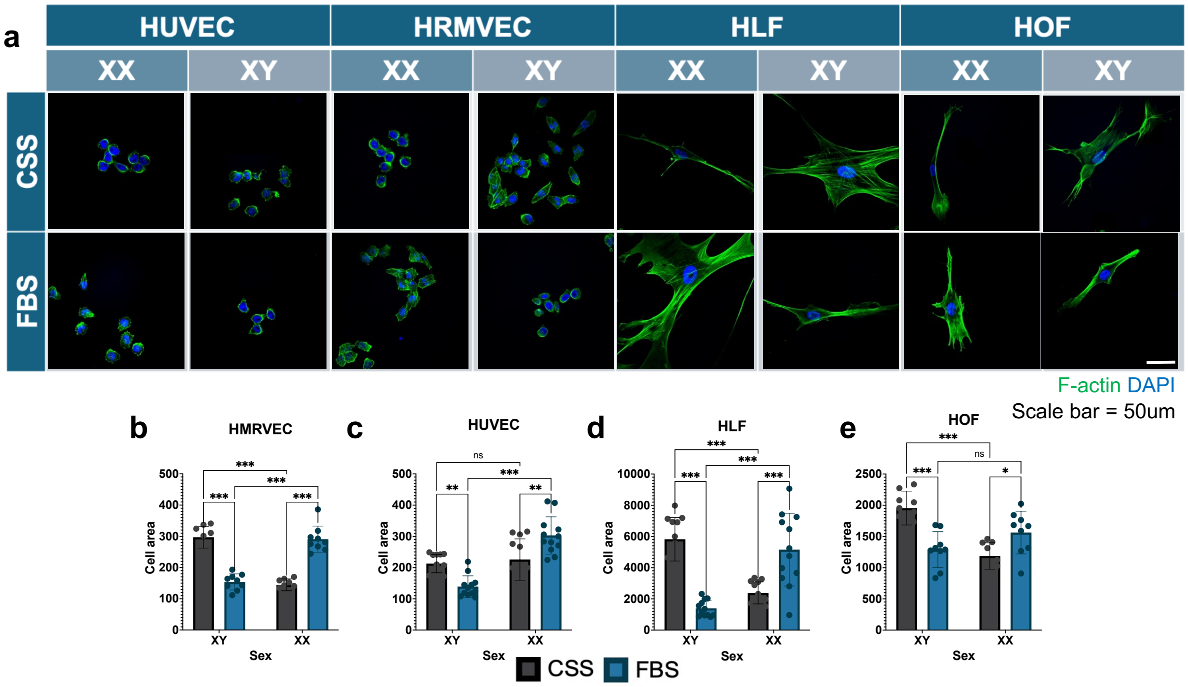

Figure S6: Sex-specific effects of FBS and CSS on cell area. **a**: Representative 60x micrographs of XX and XY endothelial cells (HUVEC, HRMVEC) and fibroblasts (HLF, HOF) grown in both FBS and CSS show decreased cytosolic spreading in XX cells CSS and XY cells in FBS. Cells stained for F-actin (green) and nuclei (DAPI). Scale bar = 50um. **b-e:** Cell area analysis shows that XY cells have greater area in CSS and XX cells have greater area in FBS. n=3 donors per sex for HRMVEC and HOF, 4 donors per sex for HLF and HUVEC, 3 wells per condition per donor, 20 cells per well (individual dots in the plots). Compared via two-way ANOVA in GraphPad Prism. * = $p<0.05$; ** = $p<0.01$; *** = $p<0.001$. Error bars represent SEM. Dots represent per well average.

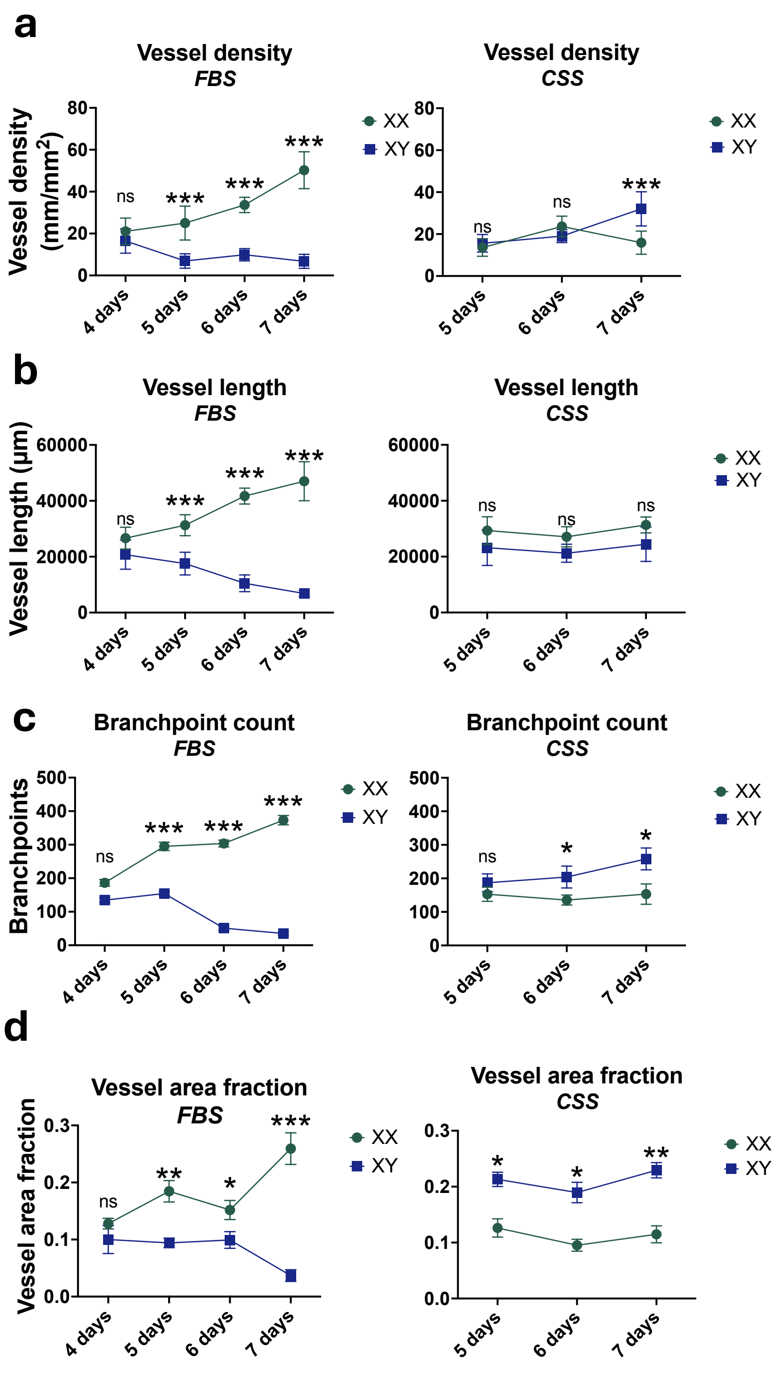

Figure S7: Sex-specific effects of FBS and CSS in the bulk vasculogenesis MPS model. Morphometric analysis revealed significant differences in vessel density (**a**), vessel length (**b**), branchpoint count (**c**), and vessel area fraction (**d**) in FBS between XX and XY devices. CSS induced significant differences in vessel densities, vessel lengths, and vessel area fractions, but not branchpoint counts. * = $p < 0.05$, ** = $p < 0.01$, *** = $p < 0.001$. All statistics were analyzed via two-way ANOVA in GraphPad Prism. Error bars represent SEM. 6 devices per condition per sex. 3 randomized pairs of HUVEC and HLF were used per sex, 2 devices per pairing.

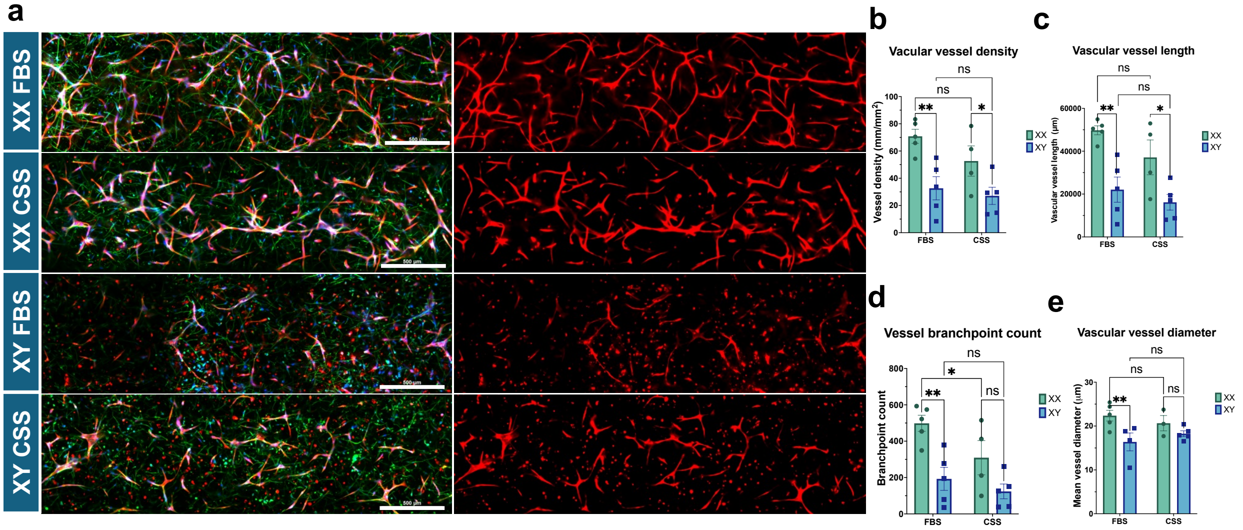

Figure S8: Sex-specific effects of FBS and CSS on initial vascular network assembly in the MPS angiogenesis model. a: Representative 3D stitched LSCM of male (XY) and female (XX) vascular networks in hormone-rich (FBS) and hormone-depleted (CSS) culture after 9 total days of culture. Endothelial cells are labelled with UEA-1 lectin (red). F-Actin in all cells is labelled with phalloidins (green). Fibroblasts are green only. Nuclei of all cells are labelled with DAPI (blue). Scale bar = 500um. **b-e:** Morphometric analysis of vascular network characteristics in the bulk vascular layer of angiogenesis devices (non-sprout vessels) (n=5). * = $p < 0.05$, ** = $p < 0.01$, *** = $p < 0.001$. All statistics were analyzed via two-way ANOVA in GraphPad Prism. Error bars represent SEM. n=5 devices per condition per sex. 2 randomized pairs of HUVEC and HLF were used per sex, 2-3 replicas per pairing. Dots represent individual devices.
